# The Graz Liver Allocation Strategy—Impact of Extended Criteria Grafts on Outcome Considering Immunological Aspects

**DOI:** 10.3389/fimmu.2020.01584

**Published:** 2020-08-04

**Authors:** Judith Kahn, Gudrun Pregartner, Alexander Avian, Daniela Kniepeiss, Helmut Müller, Peter Schemmer

**Affiliations:** ^1^General, Visceral, and Transplant Surgery, Department of Surgery, Medical University of Graz, Graz, Austria; ^2^Transplant Center Graz, Medical University of Graz, Graz, Austria; ^3^Institute for Medical Informatics, Statistics and Documentation, Medical University of Graz, Graz, Austria

**Keywords:** extended donor criteria, immunological aspects, liver transplantation, liver allocation, outcome

## Abstract

**Background:** Transplant centers are forced to use livers of extended criteria donors for transplantation due to a dramatic organ shortage. The outcome effect of extended donor criteria (EDCs) remains unclear. Thus, this study was designed to assess the impact of EDCs on outcome including immunological aspects after liver transplantation (LT).

**Patients and Methods:** Between November 2016 and March 2018, 49 patients (85.7% male) with a mean age of 57 ± 11 years underwent LT. The impact of EDCs on outcome after LT was assessed retrospectively using both MedOcs and ENIS (Eurotransplant Network Information System).

**Results:** About 80% of grafts derived from extended criteria donors. Alanine aminotransferase/aspartate aminotransferase (AST/ALT) levels elevated more than three times above normal values in organ donors was the only significant risk factor for primary dysfunction (PDF) and primary non-function (PNF)/Re-LT and early non-anastomotic biliary strictures (NAS). Balance of risk (BAR) score did not differ between EDC and non-EDC recipients. PDF (14.3% of all patients) and PNF (6.1% of all patients) occurred in 23.1% of EDC-graft recipients and in 10.0% of non-EDC-graft recipients (RR 2.31, *p* = 0.663). The 90-day mortality was 3.6%. There was no difference of early non-anastomotic biliary tract complications and biopsy proven rejections (BPR). There was no correlation of PDF/PNF with BPR and NAS, respectively; however, 66.7% of the patients with BPR also developed early NAS (*p* < 0.001).

**Conclusion:** With the Graz liver allocation strategy, excellent survival can be achieved selecting livers with no more than 2 not outcome-relevant EDCs for patients with MELD >20. Further, BPR is associated with biliary complications.

## Introduction

Organ shortage has driven transplant centers to extend their criteria for organ acceptance. Donors have become increasingly older and multi-morbid. Allocation strategies for liver transplantation (LT) as well as the acceptance criteria for donor organs in order to expand the entire pool of available organs (1, 2) are continuously being adapted. Various extended donor criteria (EDC) for each organ have been defined; however, the impact of these criteria on outcome after LT is still under debate. Apart from that, there is no definite answer to the question of how to measure the advantages and disadvantages of LT with EDC-grafts. Is it more adequate to judge the waitlist mortality, or the cumulative patient and graft survival, when assessing a LT program? What is the primary aim of LT? To make a long answer short: it is the utility, the generation of a maximum of life years through an optimized allocation of this scarce resource.

To better predict the mortality risk of patients on the waiting list, the model of end-stage liver disease (MELD) system, which is based on three laboratory values including serum creatinine, bilirubin, and international normalized ratio (INR) (laboratory (lab)MELD score), was introduced and adopted by many LT programs worldwide in order to prioritize patients for transplantation by urgency. This “sickest first” allocation policy shows conflicting results, and it has induced medical, ethical, and socio-economic debates. Several risk scores combining donor and/or recipient risk factors predicting outcome after LT have been developed, like the donor risk index (DRI, Eurotransplant [ET]-DRI), and balance of risk (BAR) score including 6 variables (donor age, recipient age, recipient MELD score, re-transplantation, pretransplant life support, and cold ischemic time), University of California, Los Angeles (UCLA), acute-on-chronic liver failure (ACLF), survival outcome following LT score (SOFT) using 18 risk factors, Pedi-SOFT and D-MELD (donor age × recipient MELD) scores, which are models for matching EDC grafts with low-risk recipients and vice versa in order to find a balance between urgency and utility and benefit. Allocation of an EDC graft to a high-risk recipient with a high MELD score should be avoided because of the risk of short-term mortality. Those patients were shown to benefit from high-quality grafts (3). Comorbidities that are not categorically evaluated in the above mentioned scores should also be exceptionally considered to accurately predict post-LT outcome, as a combination of comorbidities like age and aggravation of comorbidities like cardiovascular disease, and frailty can potentially lead to deleterious outcomes after LT.

Apart from that, a score can never replace subjective surgical experience when inspecting a graft during organ retrieval and directly prior to transplantation after having reviewed a particular recipient's condition at the time of transplant.

EDC-grafts have been widely used in the Eurotransplant (ET) region. Good results can be achieved using such liver grafts. An increased risk for biliary tract complications, primarily non-anastomotic biliary strictures (NAS), as well as vascular complications associated with the various types of EDC, as well as an potential increase of early malfunction, i.e., primary dysfunction (PPF) and primary non-function (PNF), have been reported after LT using EDC-grafts (4). Implications on acute and chronic graft rejection have been proposed (5), representing a link between the degree of ischemia reperfusion injury (IRI) and activation of innate immunity (6). EDC in LT is a hot topic. Various EDC have a different impact on outcome after LT.

Here the impact of the Graz allocation strategy (no acceptance of potentially outcome-relevant EDCs in >20 MELD recipients; i.e., >3-fold elevation of normal ranges of aspartate aminotransferase (AST) or alanine aminotransferase (ALT) or cold ischemic time (CIT > 10.5 h) on outcome after LT has been assessed considering immunological aspects in a low volume transplant center (≤40 LT/year) in a non-MELD based allocation system.

## Patients and Methods

All clinical, demographic, surgical, and post-surgical follow-up data were analyzed from all consecutive primary LT performed between November 2016 and March 2018 in a single transplant center. Based on the definition of EDC by the executive committee of the German Federal Medical Society and the ET definition the following donor criteria were assessed as EDC: donor age >65 years, ventilation >7 days, >3-fold elevation of normal ranges AST or ALT, bilirubin >3 mg/dl, peak serum sodium >165 mmol/l, biopsy-proven macrovesicular steatosis >40%, prolonged hypotensive episodes in the donor (>1 h, <60 mmHg, inotropic drug use, e.g., dopamine >14 μg/kg/min) or donor cardiac arrest, body mass index (BMI) >30, CIT >14 h, history of extrahepatic malignancy, previous drug abuse, positive hepatitis B serology (anti-hepatitis B core [HBc] antibody and/or hepatitis B surface [HBs] antigen positive) and donation after cardiac death (DCD) grafts. The concept of EDC LT was explained to the patients on the wait list for LT, and IC was obtained in all patients prior to LT except in high urgent recipients. The presence of any EDC was assessed as well as the number of EDC, if present. The following recipient criteria were assessed: demographics, indication for LT, labMELD score, post-LT laboratory parameters for liver function and liver injury (AST, ALT, alkaline phosphatase [AP], bilirubin, γ-glutamyl transferase [GGT]), PDF, PNF, ICU stay, re-LT, biliary complications within the first 3 post-operative months, vascular complications including hepatic artery thrombosis (HAT), portal vein or hepatic vein thrombosis, bleeding requiring further surgical interventions, and rejection episodes. Primary dysfunction (PDF) and primary non-function (PNF) were defined as AST and ALT >1,500 U/l and AST >2,500 U/l, respectively, during the first 72 h after LT or re-LT/graft failure (7).

The surgical technique of LT included cavo-caval end-to-side (Piggyback technique) or side-to-side anastomosis (Belghiti modified Piggyback technique). The immunosuppressive regimen was tacrolimus based together with mycophenolic acid and a cortison taper for 3 months. Induction therapy was administered in patients <40 years of age, patients suffering from autoimmune hepatitis (AIH), patients with renal insufficiency with a glomerular filtration rate of <60 ml/min, grafts from donors after cardiac death (DCD).

This retrospective analysis was based on both MedOcs and ENIS (ET Network Information System) electronic data. The study protocol has been approved by the local ethics committee, Medical University of Graz, Austria (Ethic Committee number 30-426 ex 17/18).

### Statistical Analyses

Continuous data are presented as mean ± standard deviation or median and range, as appropriate. Categorical data are presented as absolute and relative frequencies. For continuous data differences between groups were analyzed using *t*-test, Mann Whitney *U*-test. Differences in the distribution of categorical data were analyzed using χ^2^ -test or Fisher's exact test. For risk factor analysis relative risks and corresponding 95% confidence intervals (95%CI) were calculated. R version 3.4.4 and SPSS 26.0.0.0 (IBM Corp., Armonk, NY, USA) were used for these analyses.

## Results

### General Data

**Figure 1:**
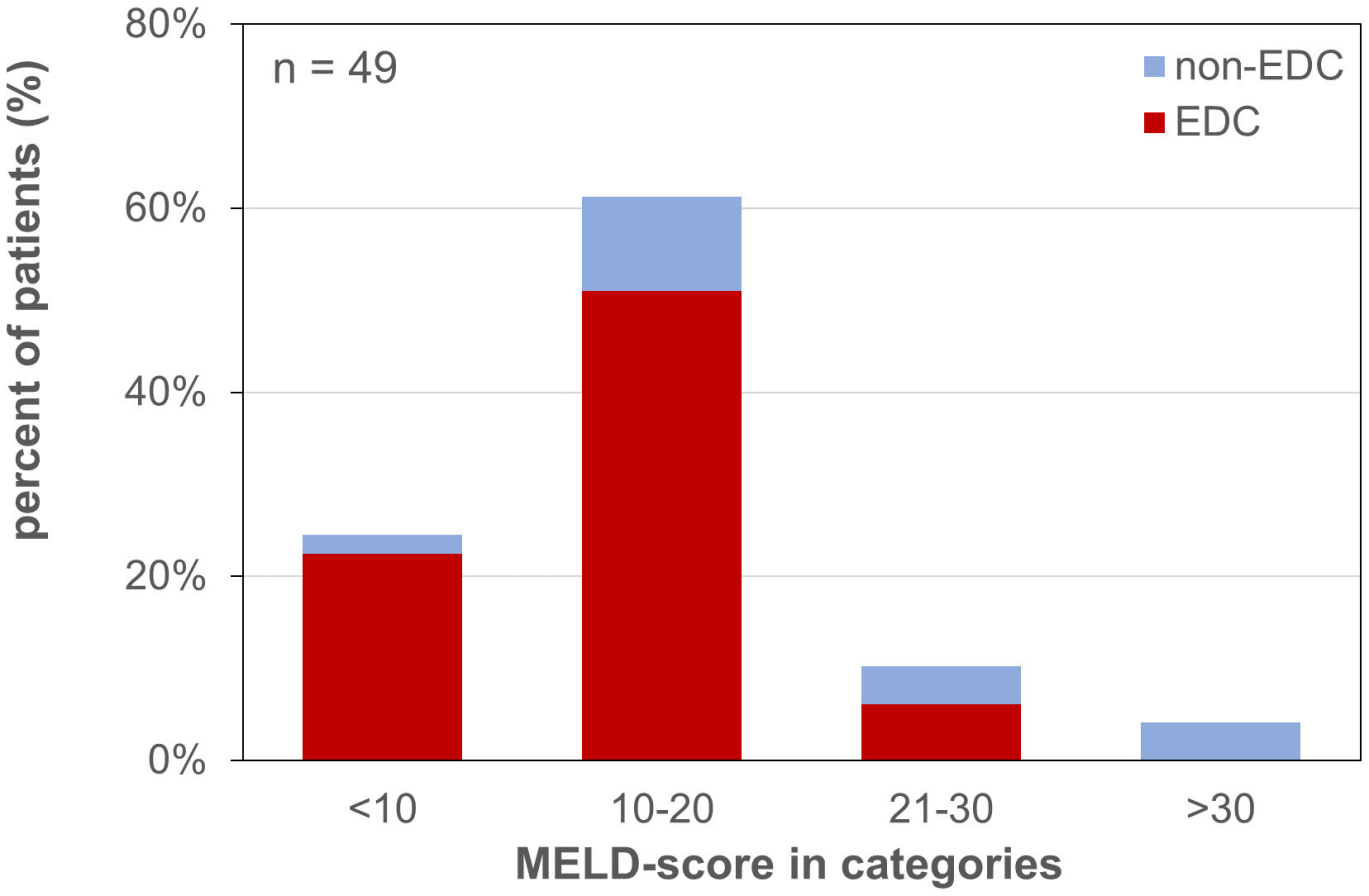
MELD-score categories of patients waiting for liver transplantation. MELD-score was categorized into 4 groups: 1: MELD <10, 2: MELD 10–20, 3: MELD 21–30, 4: MELD >30. EDC (extended donor criteria) vs. non-EDC (non-extended donor criteria) graft recipients.

**Figure 2:**
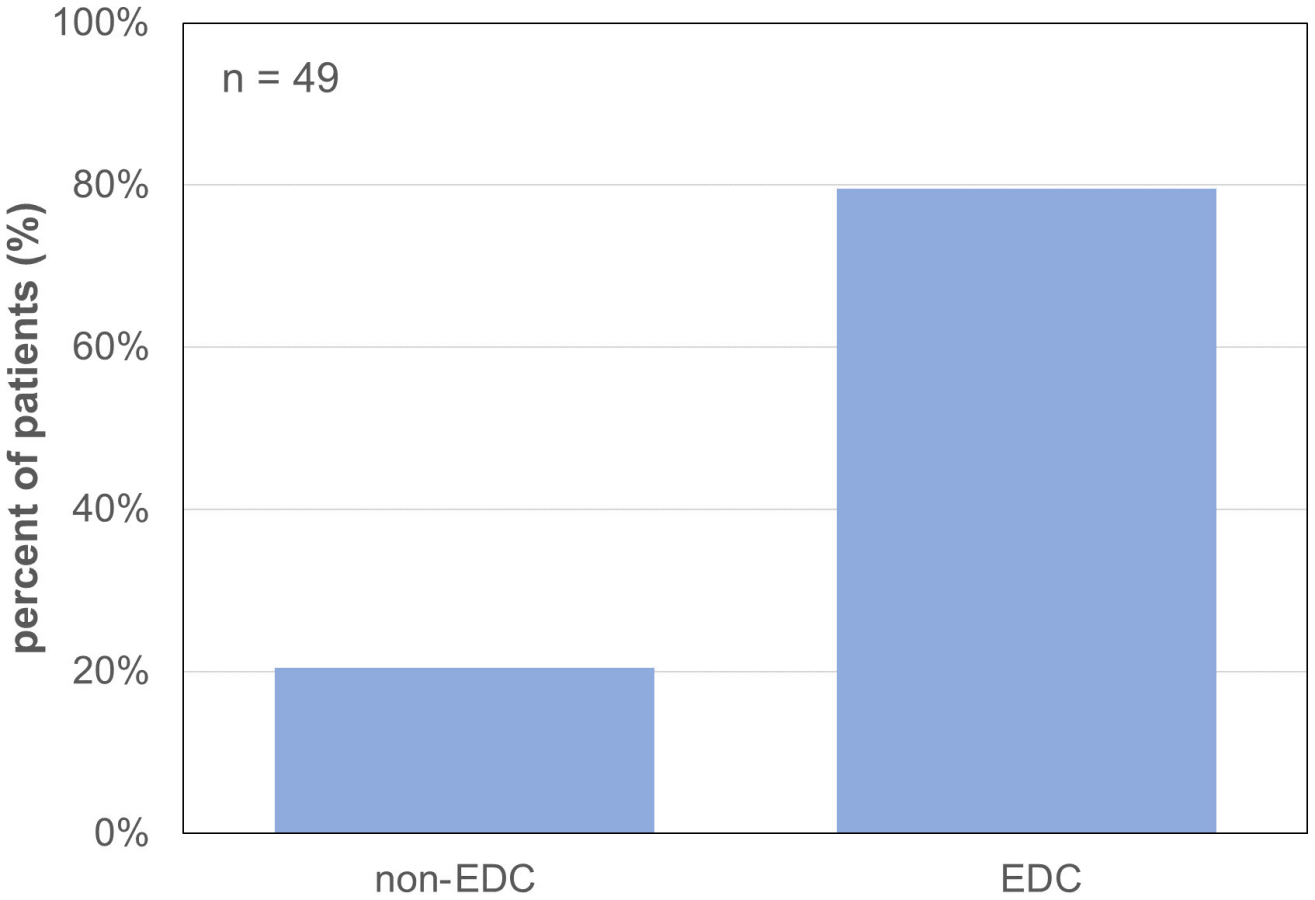
Percentage of grafts with extended donor criteria and non-extended donor criteria. 79.6% EDC (extended donor criteria) grafts and 20.4% non-EDC (non-extended donor criteria) grafts.

Forty-nine patients (85.7% male) with a mean age of 57 ± 11 years underwent LT for alcoholic liver cirrhosis (45%), hepatocellular carcinoma (HCC) (41%), primary sclerosing cholangitis (PSC) or AIH (10%), HBV-associated liver cirrhosis (2%), and acute liver failure (ALF) (2%) (Table 1). The median labMELD score of the patients was 15 (range 7–32), and 24.5% of patients presented with a labMELD score of <10, 61.2% of cases have shown a labMELD score of 10–20, 10.2% were identified with a labMELD score of 21–30, and 4.1% of patients were documented with a labMELD score of >30 (labMELD categories 1–4, Figure 1). Three patients underwent re-LT, one of which for PNF and the other 2 cases for HAT. Re-LT were excluded from further analysis, and 79.6% of grafts have shown up to three EDCs (Figure 2); the categories of EDCs are shown in Table 2. Of those, 51.3% had 1 EDC, 38.5% had 2 EDCs, and 10.2% had 3 or 4 EDCs (Table 3). No EDC existed for 20.4% of grafts. Patients were classified in EDC and non-EDC recipients. These 2 groups were comparable based on demographics, indication for LT, and labMELD score. In labMELD category 1 and 2, 16.7% of the patients (MELD score ≤ 20) received a non-EDC organ, 83.3% received an EDC organ; 57.1% of the patients in labMELD category 3 and 4 (labMELD score >20) received a non-EDC organ, 42.9% received an EDC organ with no more than 2 EDC categories.

**Table 1:** Recipient characteristics.

	**all LT**	**EDC**	**Non-EDC**
**Sex**			
Male	42	34/42 (80.9%)	8/42 (19.1%)
Female	7	5/7 (71.4%)	2/7 (28.6%)
**Age at LT [years]**	56.8 ± 11.4	59.0 ± 8.4	48.2 ± 17.0
**Indication for LT**			
Alcoholic liver disease	22	17/22 (77.2%)	5/22 (22.8%)
HCC	20	18/20 (90.0%)	2/20 (10.0%)
PSC/AIH	5	3/5 (60.0%)	2/5 (40.0%)
viral (HCV/HBV), ALF	2	1/2 (50%)	1/2 (50%)
**MELD (labMELD)**	15.2 ± 6.3	13.9 ± 4.9	20.3 ± 8.5
MELD ≤ 20	42	35/42 (83.3%)	7/42 (16.7%)
MELD > 20	7	3/7 (42.9%)	4/7 (57.1%)
**HU**			
Yes	1	0/1	1/1
No	48	39/48	9/48
**BMI**	25.9 ± 3.6	25.8 ± 3.5	26.5 ± 4.1

*Data are presented as total numbers or as means ± standard deviation*.

*LT, liver transplantation; EDC, extended donor criteria; HBV, hepatitis B virus; HCV, hepatitis C virus; HCC, hepatocellular carcinoma (either primary etiology or concomitant); ALF, acute liver failure; PSC, primary sclerosing cholangitis; AIH, autoimmune hepatitis; MELD, Model of End-stage Liver Disease; HU, high urgent*.

**Table 2:** Donor characteristics.

**Extended donor criteria *n* [%]**	**All LT**
1) Age >65 years	19 [38.8]
2) Serum transaminases (AST, ALT) >3 times normal	13 [26.5]
3) ICU/MV >7 days prior to organ procurement	9 [18.4]
4) Cardiac arrest	9 [18.4]
5) BMI >30 kg/m2	5 [10.2]
6) CIT>14 h	3 [6.1]
7) Serum Na^+^ >165 mmol/L	3 [6.1]
8) History of extrahepatic malignancy^*^	3 [6.1]
9) DCD	3 [6.1]
10) Positive hepatitis serology^*^	1 [2]

*Categories of extended donor criteria*.

*Na^+^, serum sodium; BMI, body mass index; ICU, duration of intensive care unit stay before organ procurement; MV, duration of mechanical ventilation of the donor before organ procurement; CIT, cold ischemic time*.

* *Not relevant for post-transplant graft function*.

**Table 3:** Number of functionally relevant extended donor criteria per graft.

**EDC (*n*)**	***n* = 0**	***n* = 1**	***n* = 2**	***n* = 3/4**
Grafts [%]	20.4	40.8	30.6	8.2
PNF [%]	2	4.1	0	0

*EDC, extended donor criteria; PNF, primary non-function*.

### Donor/Recipient Match

BAR-score was 6.1 (±2.4) in EDC and 7.6 (±3.2) in non-EDC recipients (n.s.) (Table 4).

**Table 4:** Balance of risk (BAR) score in extended donor criteria (EDC)/Non-EDC recipients.

	**EDC**	**Non-EDC**
BAR-score	6.1 (±2.4)	7.6 (±3.2)

*BAR-score, Balance of risk score: including 6 variables (donor age, recipient age, recipient MELD score, re-transplantation, pretransplant life support, and cold ischemic time); EDC, extended donor criteria*.

### Survival

Median follow-up time of the patients was 22 months [range 13–31 months]. One-year patient survival was 96.4% with a 90-day mortality of 3.6%. While one patient died after acute pulmonary embolism on post-operative day (POD) 7 the other cause of death was due to septic multi-organ failure. Both deaths occurred after EDC LT.

### Post-operative Data

Laboratory findings reflecting both graft injury and graft function were comparable between groups. All early (first post-operative 3 months) but one (HAT after non-EDC LT), and all late surgical re-interventions due to bleeding, vascular complication, and incisional hernia (IH) repair were performed in EDC-graft recipients. Of all EDC-graft recipients, 20.5% had 1 re-intervention, 5.1% had 2 re-interventions, and 2.6% had 3 re-interventions.

EDC LT had no significant impact on both the ICU stay and ventilation time. The median ICU stay was 2 days in both groups; however, the range of ventilation time with 1–60 days was higher in EDC-graft recipients as compared to 1–9 days after non-EDC LT; 25.6% of EDC-graft recipients requiring more than 4 days of ICU in contrast to 10.0% after non-EDC LT (RR 1.19; *p* = 0.419).

While the median ventilation time after LT was comparable after both EDC and non-EDC LT with 11 and 15 h, respectively; the range was higher after EDC LT with 5–179 h as compared to 8–65 h after non-EDC LT. Only 7.9% of EDC-graft recipients required a post-operative ventilation of more than 24 h. This is in contrast to 22.2% after non-EDC LT (RR 0.72; *p* = 0.240).

Temporary post-operative hemodialysis was necessary in 12.2% of all patients with no difference between groups (EDC-graft recipients: 12.8%, non-EDC-graft recipients: 10.0%; RR of 1.28; *p* = 1).

### Early Graft Function

**Figure 3:**
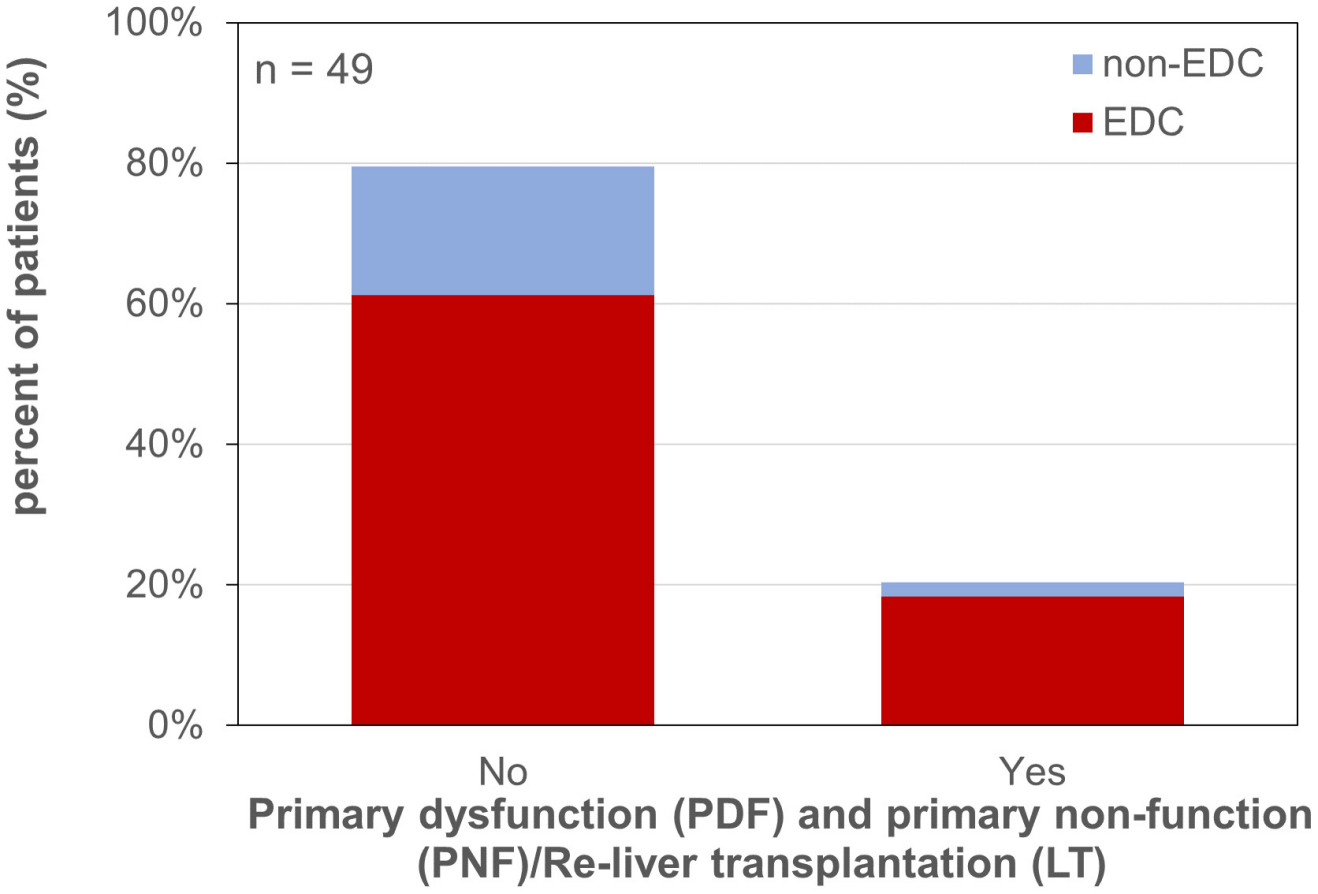
Primary dysfunction and primary non-function/re-liver transplantation. EDC (extended donor criteria) vs. non-EDC (non-extended donor criteria) graft recipients.

PDF (14.3% of all patients), PNF and the necessity for re-LT (PNF/Re-LT; 6.1% of all patients) occurred in 23.1% of cases after EDC LT and in 10.0% after non-EDC LT (RR 2.31; *p* = 0.663; Figure 3).

### Biliary Complications

**Figure 4:**
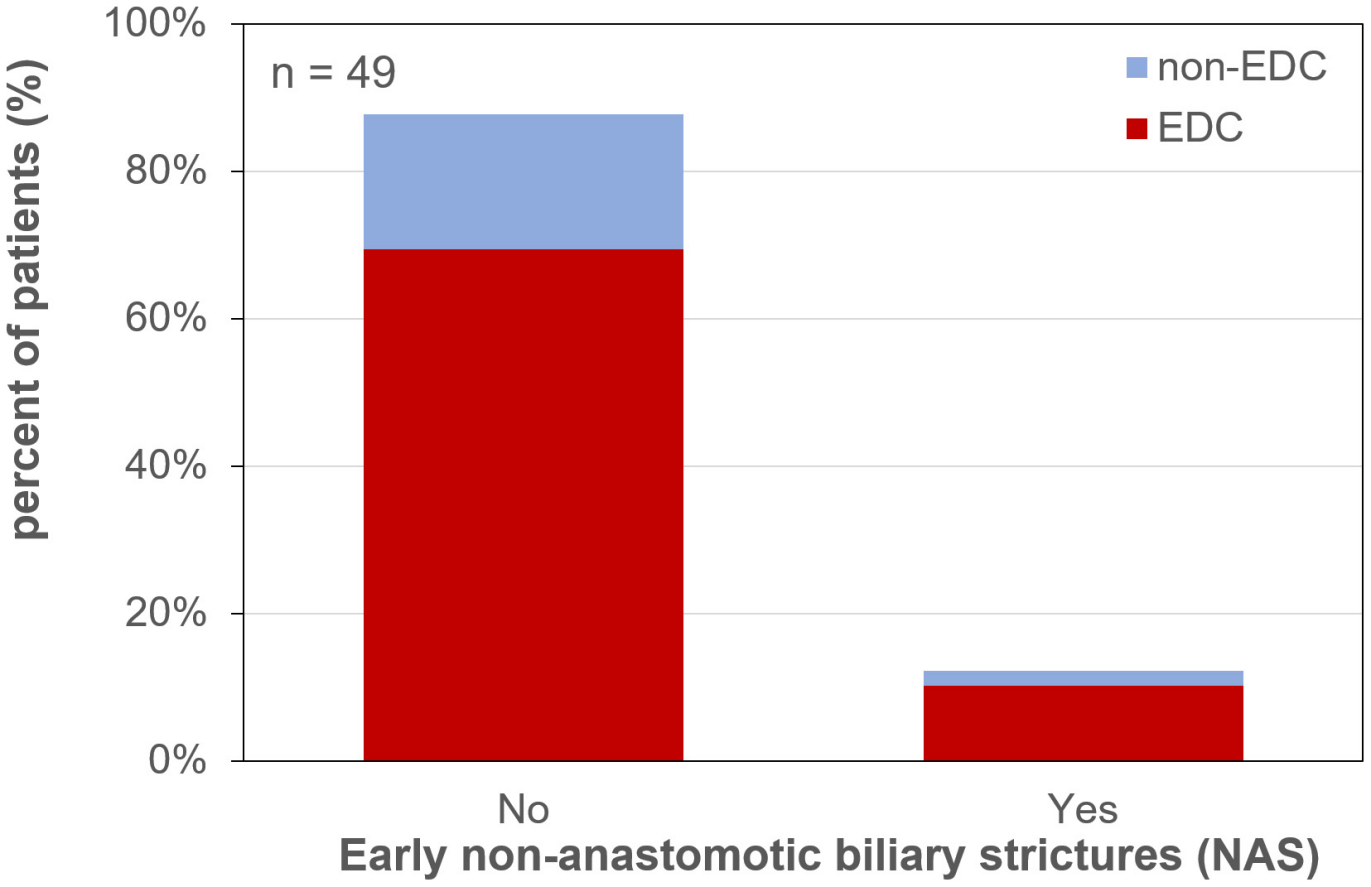
Early non-anastomotic biliary strictures after liver transplantation. EDC (extended donor criteria) vs. non-EDC (non-extended donor criteria) graft recipients.

Early NAS (12.2% of all patients during the first 3 months) occurred in 12.8% of EDC-graft recipients and in 10.0% after non-EDC LT (RR of 1.28; *p* = 1; Figure 4).

### Biopsy-Proven Acute and Chronic Rejections (BPR)

**Figure 5:**
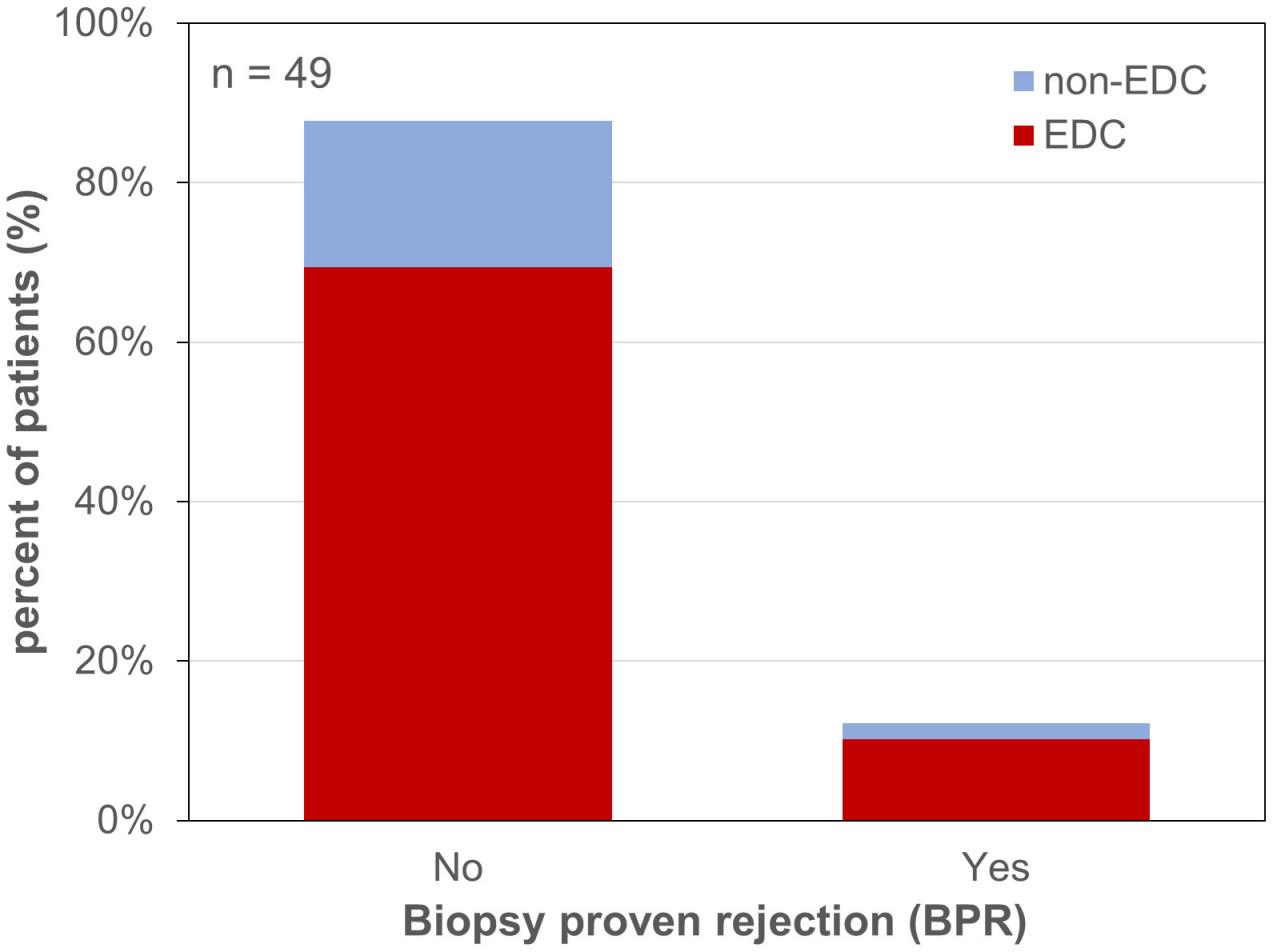
Biopsy proven rejection after liver transplantation. EDC (extended donor criteria) vs. non-EDC (non-extended donor criteria) graft recipients.

Six patients developed BPR after a median follow up of 106.5 days (6–177 days) post-LT. BPR occurred in 12.8% in EDC recipients (grafts with 1 EDC: 3 patients; grafts with 3 or 4 EDCs: 2 patients, respectively) and in 10.0% in non-EDC recipients (RR of 1.28; *p* = 1; Figure 5). There was a coincidence of BPR with PDF/PNF in 33.3% of cases (*p* = 0.588), and 66.7% of the patients with BPR also developed early NAS (*p* < 0.001).

### Immunosuppression

Intra-patient tacrolimus trough level variability within the first post-LT year did not differ between EDC- and non-EDC-graft recipients (42.5 ± 1.9% vs. 49.9 ± 10.8%, respectively; *p* = 1).

### Risk Factor Analysis

AST/ALT serum levels in organ donors more than three times increased above normal limits was a significant risk factor for PDF and PNF/Re-LT (RR 4.15, 95%CI 1.39–12.41; *p* = 0.024) as well as for early NAS (RR 5.54, 95%CI 1.62–18.99; *p* = 0.003). A CIT of > 10.5 h was the second strongest risk factor for PDF and PNF/Re-LT (RR 3.33, 95%CI 0.95–11.71) and for early NAS (RR 2.08, 95%CI 0.64–6.77).

**Figure 6:**
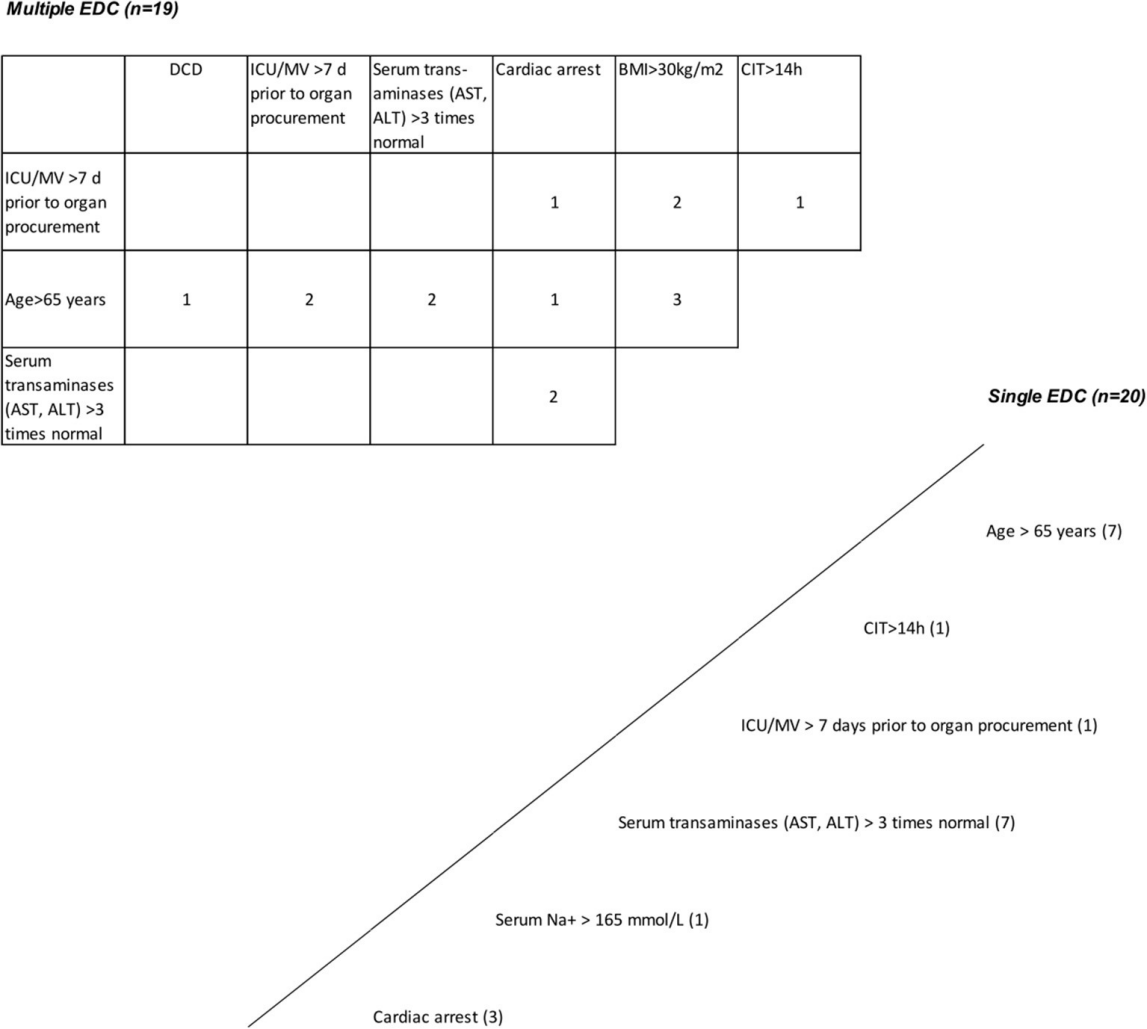
Grafts with extended donor criteria (EDCs). Single EDC—multiple EDC. A total of 49 primary cadaveric LTs were analyzed. Up to four EDCs were present in 79.6% of all grafts. Grafts with single EDC are shown below and right to the oblique line. Nineteen grafts had more than one extended criterion (table above and left to the oblique line; multiple EDCs [*n* = 19]). The number within the boxes represents how many grafts had the corresponding combined EDC. Prolonged cold ischemia time >14 h and ICU stay with ventilation >7 days, and cardiac arrest and alanine aminotransferase/aspartate aminotransferase (AST/ALT) levels >3 times normal were present in 2 grafts from donation after cardiac death (DCD) donors >65 years; AST/ALT levels >3 times normal in combination with cardiac arrest and positive hepatitis B virus (HBV) serology^*^, and in combination with hypernatremia >165 mmol/l and intensive care unit (ICU) stay with ventilation >7 days were present in 2 grafts; a history of extrahepatic malignancy^*^ in combination with donor age >65 years, cardiac arrest and hypernatremia >165 mmol/l was present in 1 graft, in combination with ICU stay with ventilation >7 days and a prolonged cold ischemic time (CIT) in another (4 grafts with 3 or more functionally relevant EDCs). ^*^Not relevant for post-transplant graft function.

**Figure 7:**
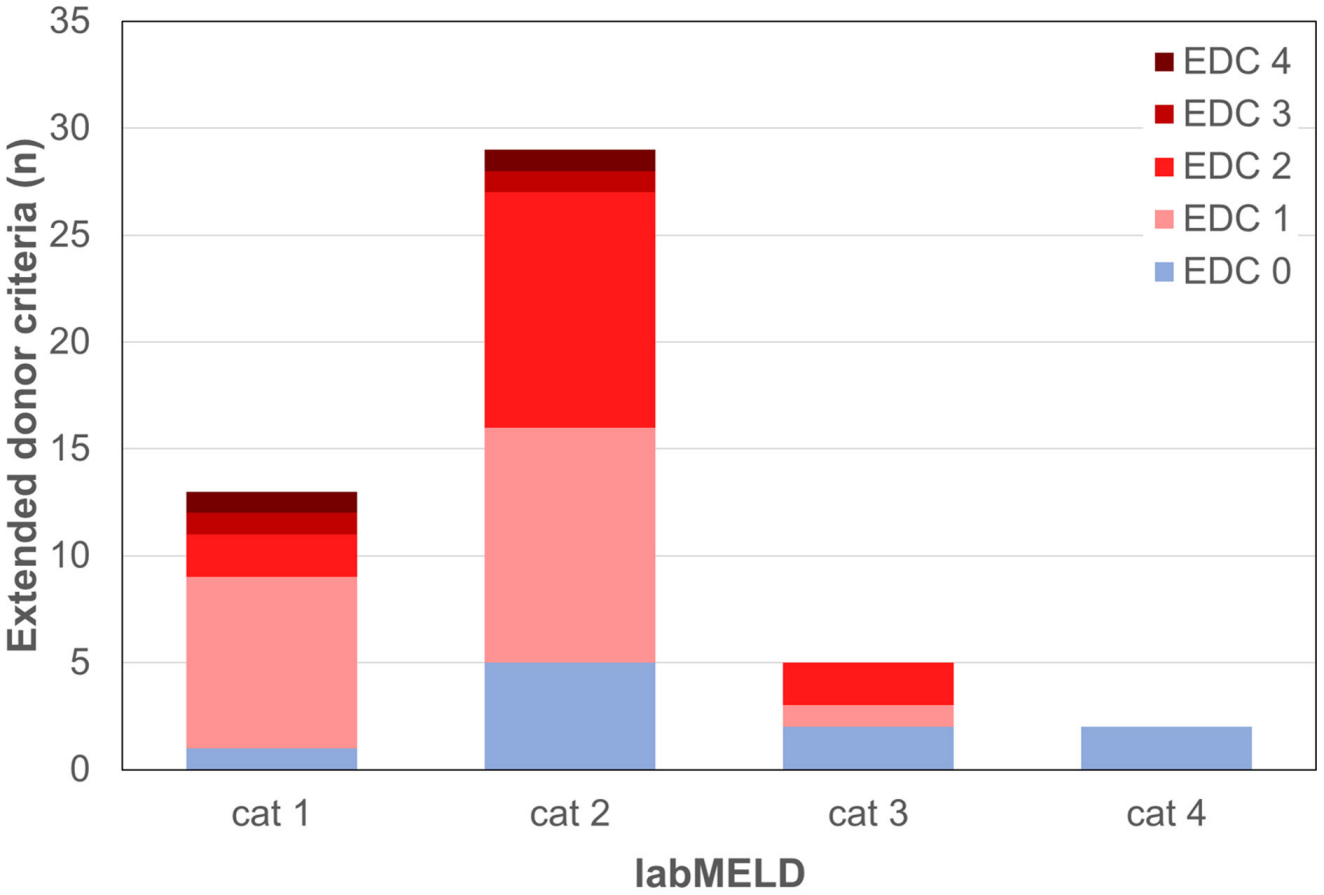
Extended donor criteria (EDCs)/laboratory model of end stage liver disease (labMELD) categories (cat 1-4). Distribution of grafts with no EDCs/EDCs (n=0-4) among recipients at different risk/labMELD category 1 (labMELD <10), category 2 (labMELD 10–20), category 3 (labMELD 21–30), and category 4 (labMELD >30). In category 3 and 4 grafts with >3-fold increased AST/ALT levels and CIT > 10.5 h were not used.

All patients with a labMELD score >20 received either non-EDC grafts or EDC grafts with no more than 2 EDCs which did not include increased AST/ALT levels or prolonged CIT (Figures 6, 7).

## Discussion

Due to the increasing lack of organs, the criteria that define donor organs suitable for LT are constantly being expanded. While older donor age or resuscitation of the donor, for example, were absolute contraindications for LT 30 years ago, today this is at most a relative contraindication. Nevertheless, survival after LT has steadily increased over the years. One-year survival is currently more than 90%, 5-year survival 80%, and 10-year survival more than 70% according to the European Liver Transplant Registry (ELTR) (8), EASL Clinical Practice Guidelines (CPG) LT (9). What are the current challenges? These are primarily early functional disorders of the graft such as PDF and PNF in 2–15% of cases (10, 11), as well as the long-term consequences of the immunosuppression (IS). The primary aim of LT is the generation of a maximum of life years through an optimized allocation of this scarce resource (12).

### EDC Definition

There is no unique definition for EDC. But, there are two categories: (i) factors directly influencing post-transplant-function and (ii) factors not influencing post-transplant-function. There was a consensus conference in 2007 on extended donor criteria, and those were defined as donor age, macrosteatosis, elevated liver enzymes, hemodynamic instability of the donor, hypernatremia, CIT, DCD, split LT, transmission of malignancy, and infections (13). Other attempts to sum up the main EDC criteria are the ET score, the German Medical Association (BÄK) score, and the UNOS definition score (14–16).

Concerning donor factors potentially influencing post-transplant graft function, one of the most important challenges is the fact that the age of donors is constantly increasing. Potential risks of LT using aged grafts are higher rates of transplant failure PNF and PDF with potentially increased mortality, and a higher degree of ischemia reperfusion injury (IRI) due to less potential to regenerate. The risk in hepatitis C positive aged grafts is even higher, as well as the damage due to longer CIT in combination with aged grafts. Some studies confirmed the negative consequences of such grafts (17–19), especially in the context of hepatitis C positivity, but many studies to date have confirmed no disadvantages for patients receiving aged liver grafts in large cohorts (up to 23,763 patients) (20, 21). Macrosteatosis of >60% of the donor liver is an unacceptable risk for graft failure, while 30–60% macrosteatosis can achieve acceptable outcomes in select donor-recipient combinations (9). The balance of risk score (BAR) is one attempt to combine the strongest donor and recipient risk predictors to generate a risk score predicting less survival for a BAR score of >18 (22). Elevated liver enzymes AST and ALT of donor livers were shown to achieve good results after LT (23), whereas elevated GGT and INR were shown to be associated with inferior results (13). Hypotensive episodes in organ donors as well as donor resuscitation were associated with non-inferior LT results (24). The need of catecholamines (norepinephrine, dopamine >10 mcg/kg/min) was shown to be a risk factor for graft failure (25). Lower patient and graft survival with donor hypernatremia >155 mEq/l was reported in several studies (26, 27), whereas most recent studies on donor hypernatremia showed no influence on patient and graft survival (28, 29). CIT of more than 8 h leads to impaired 5-year-survival after LT (7), and with each additional hour of CIT the risk for PNF increases by 1% (30).

LT after DCD has steadily increased over the years, with a DCD rate of >20% in the UK and around 6% in the United States (31). Associated risks with DCD LT include biliary tract complications (i.e., ischemic type biliary lesions [ITBL]), vascular complications like HAT, as well as PDF and PNF potentially necessitating re-LT. An increased rate of biliary tract complications of more than 30% was reported by various groups (32, 33), whereas similar 1-, 3-, and 5-year survival was reported by Kollmann et al. (34), and similar 1- and 10-year survival, but inferior 5-year-survival by Blok et al. (35) analyzing ET data. One recent study even showed better results in DCD LT with donor age <50 years and CIT <6 h than in DBD LT using grafts from donors >60 years (36).

The other donor criteria which were defined as “extended” like split LT, transmission of infections, and malignancy do not directly have a potential impact on graft function, PDF, and PNF.

### Donor/Recipient Matching

The experienced transplant surgeon is responsible for accepting the best possible match. General rules include that EDC organs shall not be used for the sickest patients, since these patients do not have any reserves to survive primary dysfunction or primary non-function. Further, according to the literature, the combination of 3 or more than 3 EDC factors decreases outcome quality after transplantation (37). The number of EDCs was higher in patients with lower labMELD scores, which is based on the opinion that a recipient in a good clinical condition can better tolerate an EDC graft than a patient with a higher labMELD score. This is in line with other publications (4, 38, 39). Hence, according to our data and other reports in the literature, patient and graft outcomes were not different (1, 2, 4, 12). The BAR-score, which is available before decision making of accepting or not an organ for a specific recipient, was reported to have the potential to detect unfavorable combinations of donor and recipient factors (22). It was also applied in this patient cohort. In this small volume center within a non-MELD-based allocation system, the MELD scores were generally low among patients on the waiting list for LT with only 14.3% of the patients with a labMELD score of >20, as were the BAR scores (6.1 [±2.4] in EDC and 7.6 [±3.2] in non-EDC recipients). According to findings in the literature (2, 4, 13, 27, 40, 41) the Graz allocation system was established avoiding outcome-relevant EDCs for high risk patients; patients with labMELD scores >20 received grafts with no more than 2 EDCs excluding >3-fold increased AST/ALT levels or prolonged CIT > 10.5 h which were most relevant for outcome after LT. Risk factor analysis revealed that AST/ALT levels elevated more than three times above normal values in organ donors was the only significant risk factor for primary dysfunction (PDF) and primary non-function (PNF)/Re-LT and early non-anastomotic biliary strictures (NAS).

### EDC Transplantation / Immunological Risk

In EDC-kidney transplantation (KT), Aubert et al. found an EDC-graft survival comparable to that of patients receiving a SDC transplant in KT recipients, whereby patients receiving EDC transplants who presented with circulating donor specific antibodies (DSA) at the time of transplantation had significantly worse allograft survival after 7 years than patients receiving EDC kidneys without circulating DSA at transplantation (44 vs. 85%). Recipients of EDC kidneys with circulating DSA showed a 5.6-fold increased risk of graft loss compared with all other transplant therapies [*p* < 0.001; (42)]. According to this large KT analysis including 6,891 patients allocation policies to avoid DSA and CIT could promote wider implementation of EDC transplantation in the context of organ shortage and improve its prognosis. No comparable results are available from LT cohorts, whereas allocation policies for EDC liver grafts have been modified accordingly. The so-called rescue allocation (RA) is one strategy for LT that has been implemented within the ET area mainly for this reason. Liver grafts are considered for RA when the regular organ allocation is declined by at least 3 centers or is averted because of donor instability or other unfavorable logistical reasons. Thus, such a donor enters a competitive or a single-recipient rescue organ offer procedure, respectively. The accepting center has the advantage to select a recipient from its own waiting list for these RA grafts (1), which is not common practice in all countries within ET.

According to the Collaborative Transplant Study (CTS) positive lymphocytotoxic T-cell crossmatches have been shown to be associated with significantly decreased graft survival in first kidney transplants performed from 1990 to 1999, but not from 2000 to 2007, in kidney re-transplants regardless of transplant period and in heart and liver transplants. Positive B-cell crossmatches were associated with significantly decreased kidney and heart, but not liver transplant survival (43). According to consensus guidelines on the testing and clinical management associated with HLA and non-HLA antibodies, a KT can be performed in the absence of a prospective crossmatch if single-antigen bead screening for antibodies to all class I and II HLA loci is negative. The presence of DSA HLA antibodies should be avoided in heart and lung transplantation and considered a risk factor for liver, intestinal, and islet cell transplantation (44).

### Biliary Complications/Immunological Link

Biliary complications after LT have a constant incidence of 10–15%. Anastomotic biliary strictures (AS) are more related to technical aspects as bile leaks, or HAT, whereas NAS are related to risk factors including immunologic, IRI, or consequences of infectious complications (45). ITBL (46) is one of the major post-operative complications accounting for up to 38% of morbidity and mortality rates of all biliary complications.

In the longer term, NAS potentially result from the use of grafts with various EDC and can be a consequence of profound IRI, as well as an increased incidence of acute and chronic rejection (4–6, 47). EDC-liver grafts are more susceptible to cold and warm IRI and develop more easily ITBL than normal livers (48), as ischemic cholangiopathy is more common with the use of DCD grafts and prolonged warm ischemic time (WIT) (49). Several studies link ITBL to various immunologically mediated processes such as AB0-incompatible liver transplants, PSC, PBC, and AIH (50).

Immunological risk factors like PBC, crossmatch positivity, and acute and chronic rejection were found to be important variables associated with the development of biliary strictures after LT in a retrospective analysis of 273 DBD LT (45), independent from IRI. An immunological component causing ITBL could be confirmed by the detection of DSA HLA antibodies in LT recipients (51).

### EDC Transplantation / Rejection

Organ age has been linked to higher acute rejection rates (52). Experimental data show that age-associated epigenetic changes that result in hypermethylation of the CpG regions or hypomethylation of the non-CpG regions (53) may increase the immunogenicity of the DNA; hypomethylation of aged DNA has been reported to induce a stronger activation of dendritic cells (DCs) compared to DNA from young donors (54). Old DCs have also been shown to secrete more inflammatory cytokines upon stimulation, possibly via decreased activation of PI3K-signaling pathways and reduced suppression of p38-MAPK activation (55). Although immunosenescence leads to an overall decline of immune function, enhanced antigen-presenting capacities have been reported (56). Older endothelial cells express higher levels of VCAM-1 and MCP-1, facilitating leukocyte adhesion and infiltration and thereby contribute to enhanced immunogenicity (57).

The compromised repairing capacity of aged organs may also play an important role for an aggravated immune response. Cell death via apoptosis is a physiological part of the aging process and older grafts contain more apoptotic cells representing a significant source of local inflammation (54, 55). As a consequence of impaired repairing capacity, old parenchymal cells express more MHC molecules (58).

Non-specific injuries like IRI, and a mechanical trauma during explantation, induce a proinflammatory milieu which can activate the innate immune response and initiate the adaptive immune response. This can be aggravated by longer CIT, also potentially leading to an increased rate of acute rejection (59, 60).

Activation and recruitment of recipient's dendritic cells (DCs) into the graft activating recipient's T cells via the indirect pathway, together with increased apoptosis and antigen presentation augmenting the immune response, represents an important link between injury and immune response (56). It has been shown that IRI enhances the immunogenicity of allograft-derived DCs via toll-like receptor 4 and nuclear factor-kappa B activation (59).

In steatotic livers, the increased volume of the hepatocytes leads to microcirculatory impairment and thereby to an increased susceptibility to IRI with an immunological impact as mentioned above.

In conclusion, the immune response against steatotic grafts and older grafts can be enhanced relative to younger grafts with cryptic self-antigens exposed during necrotic cell death involved (56).

## Conclusion

Results of our data are in accordance with previous findings, that excellent survival can be achieved with careful selection of EDC-liver grafts and appropriate recipient matching (EDC grafts for low-risk recipients and vice versa). However, there is an increased risk for biliary complications associated with the various types of EDC, and there is an indication that there may be implications in rejection, but without increased mortality risk. We also found no significant difference with respect to biliary complications, PDF/PNF, and rejection between EDC- and non-EDC-graft recipients. Altogether, the Graz allocation strategy has been proven to be safe and effective within a non-MELD based allocation system.

## Data Availability Statement

The datasets analyzed in this article are not publicly available. Requests to access the datasets should be directed to judith.kahn@medunigraz.at.

## Ethics Statement

The studies involving human participants were reviewed and approved by ethics commission of the Medical University of Graz. The patients/participants provided their written informed consent to participate in this study.

## Author Contributions

JK performed the study and wrote the manuscript. GP and AA analyzed the data. PS designed and performed the study, and wrote the manuscript. DK and HM edited the manuscript. All authors contributed to the article and approved the submitted version.

## Conflict of Interest

The authors declare that the research was conducted in the absence of any commercial or financial relationships that could be construed as a potential conflict of interest.

## Abbreviations

EDC: extended donor criteria
LT: liver transplantation
AST: alanine aminotransferase
ALT: aspartate aminotransferase
HBV: hepatitis B virus anti-HBc antibody, anti-hepatitis B core antibody HBs antigen, hepatitis B surface antigen
PDF: primary dysfunction
PNF: primary non-function
BPR: biopsy proven rejections
NAS: non-anastomotic biliary strictures
UCLA: University of California, Los Angeles
INR: international normalized ratio
MELD: model of end stage liver disease
labMELD: laboratory model of end stage liver disease
DRI: donor risk index
ET-DRI: Eurotransplant donor risk index
BAR score: balance of risk score
ACLF: acute-on-chronic liver failure
SOFT: survival outcome following liver transplantation score
Pedi-SOFT: pediatric survival outcome following liver transplantation score
BMI: body mass index
CIT: cold ischemic time
AP: alkaline phosphatase
GGT: γ-glutamyl transferase
HAT: hepatic artery thrombosis
IC: informed consent
DCD: donation after cardiac death
AIH: autoimmune hepatitis
ALF: acute liver failure
ELTR: European Liver Transplant Registry
EASL CPG: European association of the study of the liver Clinical Practice Guidelines
IS: immunosuppression
ITBL: ischemic type biliary lesions
ICU: intensive care unit
IRI: ischemia reperfusion injury
BÄK: German Medical Association
RA: rescue allocation
CTS: Collaborative Transplant Study
KT: kidney transplantation
DSA: donor specific antibodies
DBD: donation after brain death
WIT: warm ischemic time
DC: dendritic cells.
